# Lemon-Fruit-Based Green Synthesis of Zinc Oxide Nanoparticles and Titanium Dioxide Nanoparticles against Soft Rot Bacterial Pathogen *Dickeya dadantii*

**DOI:** 10.3390/biom9120863

**Published:** 2019-12-11

**Authors:** Afsana Hossain, Yasmine Abdallah, Md. Arshad Ali, Md. Mahidul Islam Masum, Bin Li, Guochang Sun, Youqing Meng, Yanli Wang, Qianli An

**Affiliations:** 1 State Key Laboratory of Rice Biology, Ministry of Agriculture Key Lab of Molecular Biology of Crop Pathogens and Insects, Zhejiang Province Key Laboratory of Biology of Crop Pathogens and Insects, Institute of Biotechnology, Zhejiang University, Hangzhou 310058, China; afsana_07@yahoo.com (A.H.); yasmeen.abdallah@mu.edu.eg (Y.A.); alibau201@gmail.com (M.A.A.); masum@bsmrau.edu.bd (M.M.I.M.); libin0571@zju.edu.cn (B.L.); 2 Department of Plant Pathology and Seed Science, Sylhet Agricultural University, Sylhet 3100, Bangladesh; 3 State Key Laboratory for Quality and Safety of Agro-products, Institute of Plant Protection and Microbiology, Zhejiang Academy of Agricultural Sciences, Hangzhou 310021, China; sungc01@sina.com; 4 General Station of Plant Protection and Quarantine of Zhejiang Province, Hangzhou 310020, China; yqmeng_77@163.com

**Keywords:** plant-based green synthesis, nanomaterials, soft rot, *Citrus limon*, *Ipomoea batatas*

## Abstract

Edible plant fruits are safe raw materials free of toxicants and rich in biomolecules for reducing metal ions and stabilizing nanoparticles. Zinc oxide nanoparticles (ZnONPs) and titanium dioxide nanoparticles (TiO_2_NPs) are the most produced consumer nanomaterials and have known antibacterial activities but have rarely been used against phytopathogenic bacteria. Here, we synthesized ZnONPs and TiO_2_NPs simply by mixing ZnO or TiO_2_ solution with a lemon fruit extract at room temperature and showed their antibacterial activities against *Dickeya dadantii*, which causes sweet potato stem and root rot disease occurring in major sweet potato planting areas in China. Ultraviolet–visible spectrometry and energy dispersive spectroscopy determined their physiochemical characteristics. Transmission electron microscopy, scanning electron microscopy, and X-ray diffraction spectroscopy revealed the nanoscale size and polymorphic crystalline structures of the ZnONPs and TiO_2_NPs. Fourier-transform infrared spectroscopy revealed their surface stabilization groups from the lemon fruit extract. In contrast to ZnO and TiO_2_, which had no antibacterial activity against *D. dadantii*, ZnONPs and TiO_2_NPs showed inhibitions on *D. dadantii* growth, swimming motility, biofilm formation, and maceration of sweet potato tuber slices. ZnONPs and TiO_2_NPs showed similar extents of antibacterial activities, which increased with the increase of nanoparticle concentrations, and inhibited about 60% of *D. dadantii* activities at the concentration of 50 µg∙mL^−1^. The green synthetic ZnONPs and TiO_2_NPs can be used to control the sweet potato soft rot disease by control of pathogen contamination of seed tubers.

## 1. Introduction

Nanoparticles (NPs) with at least one dimension in the range of 1–100 nm have high surface-to-volume ratios and display exceptional physical, chemical, and biological properties compared to their bulk counterparts. Nanotechnology dealing with the synthesis, development, and applications of NPs has brought revolutions in catalysis, cosmetic, medicine, food, agriculture, and environment technologies [1,2,3,4].

Nanoparticles were conventionally synthesized by various physical and chemical methods, such as ultrasonication, microwave irradiation, laser vaporization, wet impregnation, and sol–gel methods [5,6,7], which have the disadvantages of using expensive equipment, stringent conditions, or toxic chemicals [1,2,3,4]. Green synthesis of NPs using ecofriendly and cost-effective reducing and stabilizing materials from plants, microbes, and other natural resources and without using toxic chemicals reduces health and environmental risks at source level [1,2]. Many biomolecules in plants such as proteins, polysaccharides, amino acids, organic acids, and vitamins, and phytochemicals such as polyphenols, flavonoids, terpenoids, alkaloids, tannins, and alcoholic compounds are readily available in plant extracts and can act as reducing and stabilizing agents in green synthesis of NPs [2,3,4]. Plant extracts are easily prepared and can be scaled up for industrial production of NPs. Moreover, some studies have shown that plant extracts can reduce metal ions faster than microbes and produce more stable metal NPs than those based on microbes [2]. Edible plant fruits are obviously safe raw materials free of toxicants and rich in biomolecules for reducing metal ions and stabilizing NPs with minute possibility for production of harmful byproducts. Citrus fruits including oranges, lemons, limes, tangerines, and grapefruits are rich in various bioactive compounds like citric acid, ascorbic acid, and polyphenols and peels of the fruits contain flavonoid glycosides, carotenoids, limonoids, coumarins, sitosterols, glycosides, and volatile oil [8]. Extracts of citrus fruits or peels have been successfully used in making metal and metal oxide NPs, such as gold NPs [9,10], silver NPs (AgNPs) [11,12,13,14], zinc oxide (ZnO) NPs (ZnONPs) [15,16,17], and titanium dioxide (TiO_2_) NPs (TiO_2_NPs) [18].

ZnONPs and TiO_2_NPs are the most manufactured nanomaterials used in consumer products, such as sunscreens, toothpastes, cosmetics, and paints [19,20]. Moreover, ZnONPs and TiO_2_NPs show remarkable antibacterial activities against Gram-positive and Gram-negative pathogens to humans [18,21,22,23]. However, ZnONPs and TiO_2_NPs have rarely been used against phytopathogenic bacteria. Only a few studies have shown antibacterial activities of ZnONPs and TiO_2_NPs against phytopathogenic *Xanthomonas* [24,25,26].

Gram-negative bacteria belonging to the genera *Dickeya* and *Pectobacterium* within the family *Pectobacteriaceae* [27] are broad-host-range pathogens of plants [28]. They cause devastating soft rot diseases of numerous ornamental and vegetable plants by producing pectinases to degrade pectin in the middle lamella and primary plant cell walls, leading to maceration of plant tissues [29,30]. In the last two decades, some aggressive *Dickeya* species caused rice foot rot [31,32], maize stalk rot [33], potato blackleg and soft rot [34,35,36], sweet potato stem and root rot [37,38], banana soft rot, and sheath rot [39,40], threatening staple food security. There is a lack of crop varieties resistant to soft rot *Dickeya* and *Pectobacterium*. Meanwhile, large-scale use of effective antibiotics is no longer allowed in fields due to the risks of selection of antibiotic-resistant bacterial pathogens of humans or animals [30]. Antibacterial NPs may replace antibiotics to effectively control the soft rot bacteria.

Bacterial stem and root rot disease of sweet potato caused by *D. dadantii* recently broke out in major sweet potato planting areas in China [37,38] and requires effective approaches to control the disease. The objective of this study was to synthesize green ZnONPs and TiO_2_NPs with lemon fruit extract and determine their antibacterial activities against *D. dadantii* and potentials to control the disease.

## 2. Materials and Methods

### 2.1. Bacterial Culture

*Dickeya dadantii* strain CZ1501, which causes stem and root rot disease of sweet potato, was isolated from a diseased stem of a sweet potato plant (*Ipomoea batatas*) grown in Hangzhou, Zhejiang Province, China. *Dickeya dadantii* was cultured in the nutrient medium (glucose 2.5 g, beef extract 3 g, tryptone 10 g, and NaCl 5 g per liter, pH 7.0) with or without agar (15 g per liter).

### 2.2. Lemon Fruit Extract

Fresh lemon (*Citrus limon*) fruits bought from the supermarket were washed with tap water and Millipore water, cut into pieces, and dried at 60 °C for 10 h. Dry lemon pieces were ground into powder and mixed with Millipore water (1 g with 100 mL, Millipore, Molsheim, France), and then stirred continuously at 100 rpm at 60–70 °C for 4 h. After cooling to room temperature, the suspension was filtered through muslin cloth and then Whatman No. 1 filter paper; the extract (assumed as 10 mg∙mL^−1^; pH about 4) was used for the synthesis of NPs or stored at −80 °C.

### 2.3. Synthesis of ZnONPs and TiO_2_NPs

ZnO and TiO_2_ (analytical grade, purity ≥98%) (Sinopharm, Shanghai, China) were used for synthesis of ZnONPs and TiO_2_NPs, respectively. ZnO solution (0.5 M) and TiO_2_ solution (0.5 M) were prepared by dissolving ZnO (4.07 g) and TiO_2_ (4.00 g) separately in ethylene glycol (10 mL) (Sinopharm) and adding Millipore water to 100 mL. ZnONPs and TiO_2_NPs were separately synthesized using a protocol modified from a previous study [24]. The metal oxide solution (50 mL) was mixed with the extract of lemon fruits (50 mL) at the ratio 1:1 in flasks at 100 rpm at room temperature for 4 h and became colloid. After mixing the colloid (2 mL) with Millipore water (2 mL), the colloidal NPs were identified by ultraviolet-visible spectroscopy with a Shimadzu UV-2550 spectrometer (Shimadzu, Kyoto, Japan) from 200 to 800 nm at 1 nm resolution. The colloidal NPs were centrifuged at 27,200 *g* for 10 min and the pellets were washed with Millipore water and then freeze-dried with an Alpha 1-2 LDplus (Martin Christ GmbH, Osterode am Harz, Germany). The freeze-dried NPs were stored at −80 °C or prepared as stock solutions (50 mg∙mL^−1^) for further analyses.

### 2.4. Characterization of ZnONPs and TiO_2_NPs

Dry lemon powder, ZnONP powder, and TiO_2_NP powder were analyzed by Fourier transform infrared (FTIR) spectroscopy to detect groups responsible for synthesis and stabilizing ZnONPs and TiO_2_NPs as previously described [41]. Bruker infrared table (Bruker Optics Inc. Billerica, MA, USA) and LibreTexts infrared spectroscopy absorption table (https://chem.libretexts.org/) were used to interpret the FTIR spectra. The elements of ZnONPs and TiO_2_NPs were detected by energy dispersive X-ray spectroscopy [41]. The size and morphology and the crystalline nature of ZnONPs and TiO_2_NPs were observed and analyzed by transmission electron microscopy (TEM), scanning electron microscopy, and X-ray diffraction spectroscopy [41].

### 2.5. Determination of Antibacterial Activities of Nanoparticles

*Dickeya dadantii* CZ1501 grown to mid-exponential phase was adjusted with the nutrient broth to about 5 × 10^8^ CFU∙mL^−1^ before use.

Antibacterial activities against *D. dadantii* were first detected by the diffusion assay with agar plates [41]. *Dickeya dadantii* suspension was inoculated into the nutrient agar to 10^7^ cells∙mL^−1^. Wells (7 mm in diameter) were made in the agar plates with sterilized steel punchers. Fifty microliters of lemon fruit extract (10 mg∙mL^−1^), ZnO (0.5 M), TiO_2_ (0.5 M), ZnONP (50 µg∙mL^−1^), and TiO_2_NP (50 µg∙mL^−1^) were loaded into the wells and incubated at 30 °C for 24 h. Antibacterial activities were determined by the diameters of the clearing zones formed around the wells.

Antibacterial activities against *D. dadantii* growth by ZnONPs and TiO_2_NPs at different concentrations were further determined in nutrient broth [41]. Lemon fruit extract, ZnO, or TiO_2_ was added into nutrient broth to a final concentration of 50 µg∙mL^−1^. ZnONPs or TiO_2_NPs was added into nutrient broth to final concentrations of 12, 25, and 50 µg∙mL^−1^. *Dickeya dadantii* suspension (100 μL) was inoculated into the nutrient broth (5 mL) (1 × 10^7^ CFU∙mL^−1^) without (control) or with lemon fruit extract, ZnO, TiO_2_, ZnONPs, or TiO_2_NPs and grown at 200 rpm and 30 °C for 24 h. Optical density at 600 nm of the cultures was measured using a SpectraMax spectrophotometer (Molecular Devices, Sunnyvale, CA, USA) [41].

*Dickeya dadantii* suspension in nutrient broth (1 × 10^7^ CFU∙mL^−1^) without or with lemon fruit extract, ZnO, TiO_2_, ZnONPs, or TiO_2_NPs prepared as described above was transferred into wells (200 μL of suspension in each well) of 96-well microplates (Corning-Costar Crop., Corning, NY, USA) and incubated at 30 °C for 24 h. Biofilm formed by *D. dadantii* was stained by crystal violet and quantified by absorbance at 590 nm as previously described [41].

The swimming motility of *D. dadantii* was determined with semisolid nutrient agar (0.3% (*w/v*)) [41]. Lemon fruit extract, ZnO, or TiO_2_ was added into the semisolid nutrient agar to the final concentration of 50 µg∙mL^−1^. ZnONPs or TiO_2_NPs was added into the semisolid nutrient agar to final concentrations of 12, 25, and 50 µg∙mL^−1^. *Dickeya dadantii* suspension (5 µL) was spotted onto the center of the semisolid nutrient agar plates and incubated at 30 °C for 48 h. The diameters of the halo-like colonies of *D. dadantii* were measured [41].

In vivo antibacterial activity against *D. dadantii* was determined with sweet potato tuber slices [41]. Sweet potato tubers were surface-sterilized with 70% (*v*/*v*) ethanol, washed with sterile distilled water, and cut into slices (10 mm in thickness). The tuber slices were also surface-sterilized with 70% (*v*/*v*) ethanol, washed with sterile distilled water, and immersed in distilled water (control), lemon fruit extract (50 µg∙mL^−1^), ZnO solution (50 µg∙mL^−1^), TiO_2_ solution (50 µg∙mL^−1^), ZnONP solutions (12, 25, and 50 µg∙mL^−1^), or TiO_2_NP solutions (12, 25, and 50 µg∙mL^−1^) for 1 h, and then air-dried in Petri dishes for 1 h. Afterwards, *D. dadantii* suspension (5 µL) was spotted in punctures at the center of the tuber slices and incubated at 30 °C for 24 h. Diameters of the maceration zones around the punctures were measured [41].

### 2.6. Transmission Electron Microscopy on Dickeya dadantii Cell Structure

The effect of ZnONPs and TiO_2_NPs on *D. dadantii* cell structure was determined by TEM. *Dickeya dadantii* suspension (100 μL) was inoculated into the nutrient broth (5 mL) (1 × 10^7^ CFU∙mL^−1^) without (control) or with ZnONPs (50 µg∙mL^−1^) or TiO_2_NPs (50 µg∙mL^−1^) and grown at 200 rpm and 30 °C for 4 h. *Dickeya dadantii* cells were harvested and prepared for TEM as previously described [41].

### 2.7. Statistical Analysis

All data in each experiment were subjected to one-way analysis of variance and means were compared by the Duncan’s multiple range test using the SPSS software version 16 (SPSS, Chicago, IL, USA). The significance was set at *p* < 0.05.

## 3. Results

### 3.1. Characterization of ZnONPs and TiO_2_NPs Synthesized with Lemon Fruit Extract

**Figure 1:**
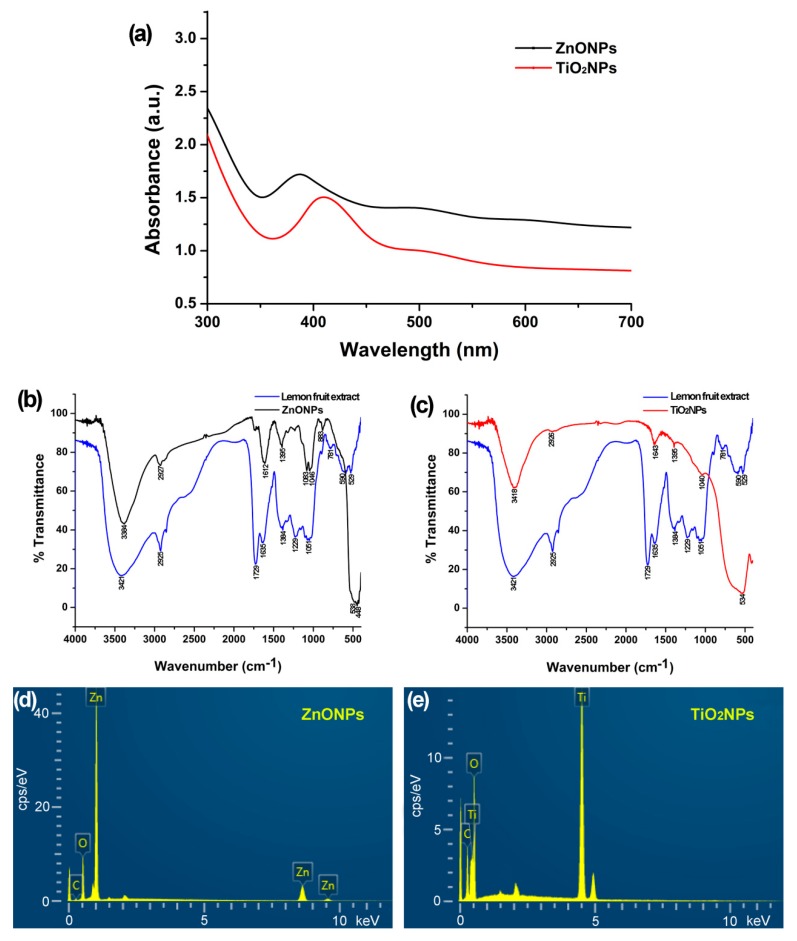
Physicochemical characteristics of zinc oxide nanoparticles (ZnONPs) and titanium dioxide nanoparticles (TiO_2_NPs) synthesized with lemon fruit extract. (**a**) ultraviolet–visible absorption spectrum of the colloidal ZnONPs and TiO_2_NPs; (**b**,**c**) Fourier transform infrared spectrum detecting groups in the lemon fruit extract and the green synthetic ZnONPs and TiO_2_NPs; (**d**,**e**) energy dispersive spectrum showing the predominance of Zn and O elements in the ZnONPs and Ti and O elements in the TiO_2_NPs.

ZnONPs and TiO_2_NPs were synthesized by constant mixing of ZnO or TiO_2_ solution with the lemon fruit extract at room temperature. ZnONPs displayed a characteristic surface plasmon resonance peak at 388 nm in the range of 350–420 nm (Figure 1a) determined by ultraviolet-visible spectroscopy [24]. TiO_2_NPs displayed a characteristic surface plasmon resonance peak at 410 nm in the range of 360–450 nm (Figure 1a) [23].

The FTIR spectrum of the ZnONPs (Figure 1b) shows major absorption bands of the Zn–O bond at 448 cm^−1^ and 538 cm^−1^ [24,42], O–H stretching of alcohol and carboxylic acid and N–H stretching of amine around 3384 cm^−1^, C–H stretching of alkane and aldehyde around 2927 cm^−1^, C=C stretching of alkene and N–H bending of amine around 1612 cm^−1^, O–H bending of alcohol around 1395 cm^−1^, C–N stretching of amine and C–O stretching of alcohol around 1083 cm^−1^ and 1046 cm^−1^, and minor absorption bands of C=O stretching about 1700–1800 cm^−1^ including carboxylic acid and C=C bending about 750–850 cm^−1^. The FTIR spectrum of the TiO_2_NPs (Figure 1c) shows major absorption bands of the Ti–O bond around 534 cm^−1^ [43], O–H stretching of alcohol and carboxylic acid and N–H stretching of amine around 3418 cm^−1^, C–H stretching of alkane and aldehyde around 2926 cm^−1^, C=C stretching of alkene and N–H bending of amine around 1643 cm^−1^, O–H bending of alcohol around 1395 cm^−1^, C–N stretching of amine or C–O stretching of alcohol around 1040 cm^−1^. The absorption bands other than the Zn–O and Ti–O bonds in the FTIR spectra of ZnONPs and TiO_2_NPs appeared in the FTIR spectrum of the dry lemon powder (Figure 1b,c), indicating that the functional groups on the NPs are from the lemon fruit extracts.

Energy dispersive X-ray spectroscopy determined Zn (77.77 wt%), O (17.51 wt%), and C (3.81 wt%) in the ZnONP powder (Figure 1d) and Ti (50.22 wt%), O (39.85 wt%), and C (9.75%) in the TiO_2_NP powder (Figure 1e).

**Figure 2:**
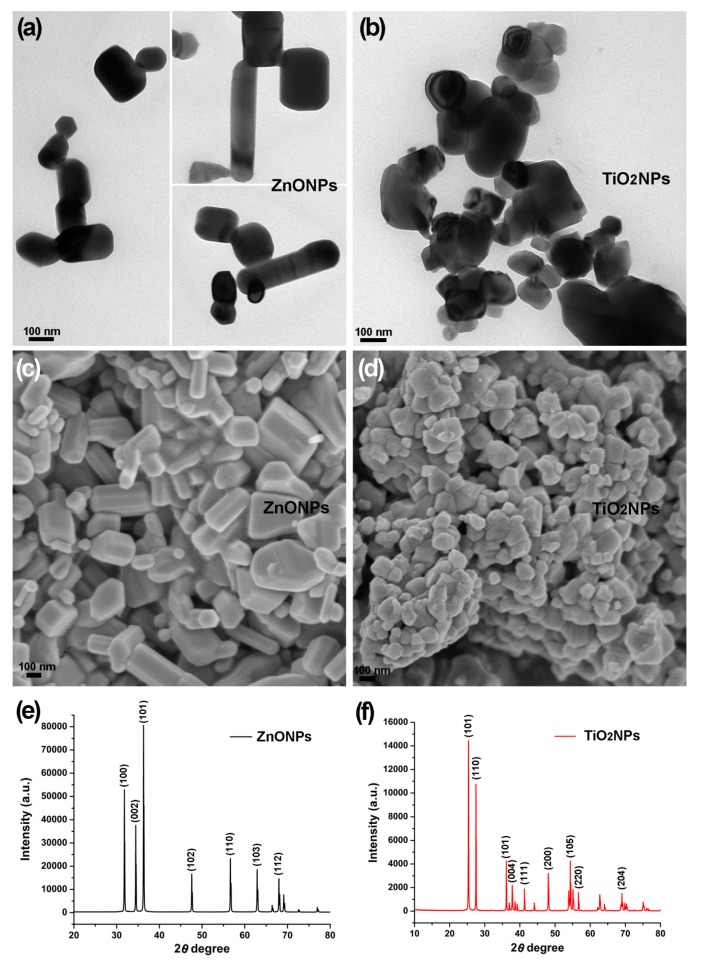
Size and morphology of zinc oxide nanoparticles (ZnONPs) and titanium dioxide nanoparticles (TiO_2_NPs) synthesized with lemon fruit extract. (**a**,**b**) Transmission electron microscopic view of polymorphic ZnONPs and TiO_2_NPs with dimensions of 20–200 nm; (**c**,**d**) scanning electron microscopic view of polymorphic ZnONPs and TiO_2_NPs; (**e**) X-ray diffraction pattern indicating crystalline wurtzite structures of the ZnONPs; (**f**) X-ray diffraction pattern indicating crystalline anatase structures and rutile structures of the TiO_2_NPs.

Electron microscopy revealed polymorphic ZnONPs (Figure 2a,c) and TiO_2_NPs (Figure 2b,d) in dimensions of 20–200 nm synthesized with the lemon fruit extract. ZnONPs displayed cuboid, hexagonal prism, thin rods, near-spheroid, and irregular shapes (Figure 2a,c) while TiO_2_NPs displayed near-spheroid and irregular shapes (Figure 2b,d); ZnONPs and TiO_2_NPs were in agglomeration after sample preparation for scanning electron microscopy (Figure 2c,d), as previous studies have shown [23,24]. The X-ray diffraction analysis confirmed the nanoscale size and crystalline nature of the ZnONPs and TiO_2_NPs. The X-ray diffraction pattern of the ZnONPs showed the characteristic Bragg reflection peaks at 2θ values of 31.81° (1 0 0), 34.46° (0 2 2), 36.20° (1 0 1), 47.58° (1 0 2), 58.64° (1 1 0), 62.90° (1 0 3), and 67.99° (1 1 2) (Figure 2e). This X-ray diffraction pattern indicating the hexagonal wurtzite structures of ZnO was identified by the standard powder diffraction card (JCPDS Card) no. 36-1451 in the Joint Committee on Powder Diffraction Standards library. The X-ray diffraction analysis revealed the mixture of anatase TiO_2_NPs and rutile TiO_2_NPs. The five characteristic reflection peaks at 2θ values of 25.34° (1 0 1), 37.80° (0 0 4), 48.09° (2 0 0), 54.35° (1 0 5), and 62.94° (2 0 4) (Figure 2f) indicated the anatase TiO_2_NPs (JCPDS Card no. 21-1272) while the four characteristic reflection peaks at 2θ values of 27.47° (1 1 0), 36.11° (1 0 1), 41.27° (1 1 1), and 56.65° (2 2 0) (Figure 2f) indicated the rutile TiO_2_NPs (JCPDS Card no. 21-1276). The average particle sizes of the ZnONPs (60.8 nm) and TiO_2_NPs (41.5 nm) were calculated using the Debye–Scherrer formula: *D* = *Kλ*/(*βCosθ*), where *D* is the average particle size, *K* is the Scherrer constant (0.9), *λ* is the X-ray wavelength (0.15406 nm), *β* is the full width at half maximum of the X-ray diffraction peak, and *θ* is the Bragg angle.

### 3.2. Antibacterial Activity of ZnONPs and TiO_2_NPs Against Dickeya dadantii

**Figure 3:**
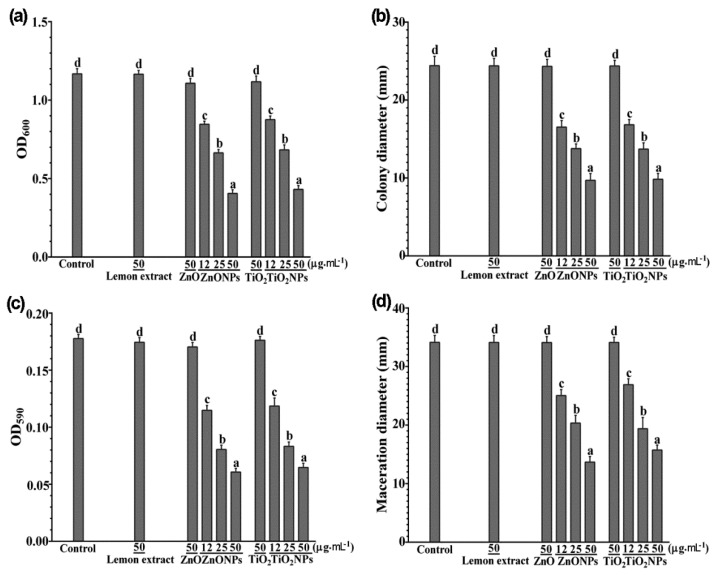
Antibacterial activity against *Dickeya dadantii* by zinc oxide nanoparticles (ZnONPs) and titanium dioxide nanoparticles (TiO_2_NPs) synthesized with lemon fruit extract. (**a**) *Dickeya dadantii* growth in liquid nutrient broth containing lemon extract (50 µg∙mL^−1^), ZnO (50 µg∙mL^−1^), ZnONPs (12, 25, or 50 µg∙mL^−1^), TiO_2_ (50 µg∙mL^−1^), or TiO_2_NPs (12, 25, or 50 µg∙mL^−1^) indicated by optical density at 600 nm (OD_600_); (**b**) *Dickeya dadantii* swimming motility indicated by diameters of halo-like colonies formed on semisolid nutrient media with lemon extract, ZnO, ZnONPs, TiO_2_, or TiO_2_NPs; (**c**) crystal violet absorbance at 590 nm (OD_590_) indicating biofilms formed by *D. dadantii* with lemon extract, ZnO, ZnONPs, TiO_2_, or TiO_2_NPs; (**d**) diameters of maceration tissues generated by *D. dadantii* in sweet potato tuber slices after 1 h immersing in lemon extract, ZnO, ZnONPs, TiO_2_, or TiO_2_NPs. Mean values with standard errors (vertical bars) are presented; the different letters on the vertical bars indicate significant difference between treatments (*p* < 0.05).

Lemon fruit extract (10 mg∙mL^−1^), ZnO (0.5 M), and TiO_2_ (0.5 M), the raw materials for synthesis of ZnONPs and TiO_2_NPs, did not generate clearing zones around them in the nutrient agar containing *D. dadantii* cells and thus did not inhibit the growth of *D. dadantii*. In contrast, the products ZnONPs (50 µg∙mL^−1^) and TiO_2_NPs (50 µg∙mL^−1^) generated clearing zones with diameters of 30.0 ± 0.7 mm and 28.5 ± 0.5 mm, respectively, around the wells (including the well diameter 7 mm) and thus inhibited the growth of *D. dadantii* or killed *D. dadantii*. In nutrient broth, ZnONPs and TiO_2_NPs significantly inhibited *D. dadantii* growth and the extents of inhibition increased with the increase of the concentrations (12, 25, and 50 µg∙mL^−1^) of ZnONPs and TiO_2_NPs, whereas lemon fruit extract, ZnO, or TiO_2_ (50 µg∙mL^−1^) did not inhibit *D. dadantii* growth (Figure 3a). ZnONPs and TiO_2_NPs inhibited *D. dadantii* growth at similar extents.

*Dickeya dadantii* grew and swam in the semisolid nutrient medium and formed a halo-like colony about 24 mm in diameter after 48 h (Figure 3b). Lemon fruit extract, ZnO, or TiO_2_ (50 µg∙mL^−1^) did not inhibit *D. dadantii* growth and swimming in the semisolid medium, whereas ZnONPs and TiO_2_NPs significantly inhibited *D. dadantii* growth and swimming and the extents of inhibition increased with the increase of the concentrations of ZnONPs and TiO_2_NPs (Figure 3b). ZnONPs and TiO_2_NPs inhibited *D. dadantii* growth and swimming motility at similar extents, about 31–32%, 44%, and 60% at the concentrations of 12, 25, and 50 µg∙mL^−1^, respectively.

*Dickeya dadantii* cells formed biofilms on the surface of the polystyrene microplate wells during the 24 h incubation. Lemon fruit extract, ZnO, or TiO_2_ (50 µg∙mL^−1^) did not inhibit the biofilm formation, whereas ZnONPs and TiO_2_NPs significantly inhibited the biofilm formation and the extents of inhibition increased with the increase of the concentrations of ZnONPs and TiO_2_NPs (Figure 3c). ZnONPs and TiO_2_NPs inhibited *D. dadantii* biofilm formation at similar extents, about 34–37%, 54–55%, and 64–66% at the concentrations of 12, 25, and 50 µg∙mL^−1^, respectively.

*Dickeya dadantii* degraded plant cell walls of sweet potato tuber cells and generated maceration zones about 34 mm in diameter in the sweet potato slices at 24 h after inoculation (Figure 3b). Lemon fruit extract, ZnO, or TiO_2_ (50 µg∙mL^−1^) did not inhibit the tissue maceration by *D. dadantii*, whereas ZnONPs and TiO_2_NPs significantly inhibited the tissue maceration and the extents of inhibition increased with the increase of the concentrations of ZnONPs and TiO_2_NPs (Figure 3d). ZnONPs and TiO_2_NPs inhibited the tissue maceration at similar extents, about 21–27%, 40–43%, and 54–60% at the concentrations of 12, 25, and 50 µg∙mL^−1^, respectively. The in vivo inhibition of tissue maceration in the sweet potato tuber was consistent with the in vitro inhibition of *D. dadantii* growth, swimming motility, and biofilm formation.

**Figure 4:**
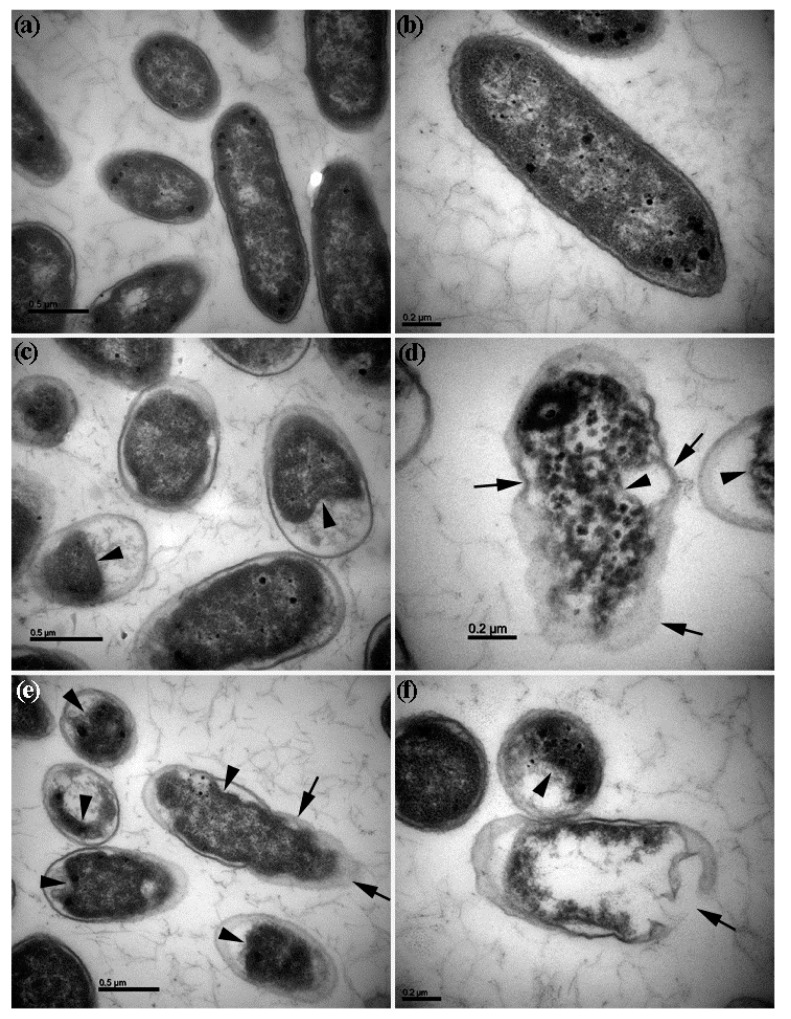
Transmission electron microscopic view of *Dickeya dadantii* cells. (**a**,**b**) *Dickeya dadantii* cells grown in nutrient broth without treatment have intact cell envelopes and dense cytoplasm filled in the cells; (**c**,**d**) *Dickeya dadantii* cells grown in nutrient broth with zinc oxide nanoparticles (ZnONPs) (50 µg∙mL^−1^) for 4 h; (**e**,**f**) *Dickeya dadantii* cells grown in nutrient broth with titanium dioxide nanoparticles (TiO_2_NPs) (50 µg∙mL^−1^) for 4 h; arrows point to distortion and disintegration of cell envelopes. Arrowheads point to shrunken, agglomerated, or collapsed cytoplasm.

Transmission electron microscopy revealed the morphological changes of *D. dadantii* cells after growing in the nutrient broth with ZnONPs and TiO_2_NPs (50 µg∙mL^−1^). *-Dickeya dadantii* cells grown without NPs had intact cell envelopes and dense cytoplasm filled in the cells (Figure 4a,b). After 4 h growth with NPs, cell envelopes of many *D. dadantii* cells became distorted and disintegrated while cytoplasm became shrunken, agglomerated, and collapsed (Figure 4c–f), leading to cell death.

## 4. Discussion

We made ZnONPs and TiO_2_NPs separately by mixing ZnO or TiO_2_ dissolved in ethylene glycol and water with the lemon fruit extract at room temperature. Energy dispersive X-ray spectroscopy on the NPs revealed C element from the lemon fruit extract. Fourier transform infrared spectroscopy revealed that the functional groups on the surface of the ZnONPs and TiO_2_NPs and responsible for stabilization of the ZnONPs and TiO_2_NPs were from the lemon fruit extract. The major O–H and N–H groups are associated with carboxylic acids such as citric acid and ascorbic acid [13], and amines such as free amino acids and proteins, which are rich in lemon fruits [8]. The O–H groups of alcohol may be from polyols in the lemon fruit extract and the solvent ethylene glycol [44]. Citric acid has three carboxylate groups and is able to form stable complexes with metal ions. Citric acid has been used as a reducing and stabilizing agent in synthesis of a wide range of nanomaterials to control both the size and morphology of the nanomaterials [13,45,46]. Ethylene glycol with two hydroxyl groups has a relatively strong reducing powder and high boiling point, and has been widely used in polyol synthesis of metal nanomaterials [44,47]. Unlike TiO_2_, ZnO is not stable in acidic solutions [48]. Dissolution of ZnO to Zn^2+^ may occur after mixing ZnO solution with the lemon fruit extract (pH 4.0) while Zn^2+^ may be chelated by citrate through two carboxyl groups and one hydroxyl group and form a pentabasic ring and a hexahydric ring [49]. Perhaps, esterification of citric acid and ethylene glycol and binding between esters and Zn^2+^ may occur in the mixture and stabilize Zn^2+^ [16]. Citric acid may also reduce the surface tension of ZnO and TiO_2_ solutions and lower the energy needed to form the ZnO and TiO_2_ crystals [49]. Together, multiple carboxylic acids, amino acids, and polyols may lead to the formation of the polymorphic ZnONPs and TiO_2_NPs.

In contrast to ZnO and TiO_2_ with no antibacterial activity against *D. dadantii*, ZnONPs and TiO_2_NPs showed distinct antibacterial activities against *D. dadantii* growth, swimming motility, biofilm formation, and maceration of sweet potato tuber slices. ZnONPs and TiO_2_NPs showed similar extents of antibacterial activities against *D. dadantii* and an increase of antibacterial activities with the increase of NP concentrations, and inhibited about 60% of *D. dadantii* growth, swimming motility, biofilm formation, and maceration of tuber slices at the concentration of 50 µg∙mL^−1^.

The distinct antibacterial activities of metal and metal oxide NPs were achieved by their smaller sizes and larger surface-area-to-mass ratios and generation of oxidative stress on bacterial cells [21,50]. The smaller size and larger surface area lead NPs to easily adsorb bacterial cells and a higher proportion of atoms on the particle surface, and enhance the ability to pass through membranes and the interfacial reactivity to directly interfere with cell envelope functions. Metal oxides like ZnO and TiO_2_ have known photo-oxidizing and photocatalytic activities and generate reactive oxygen species (ROS). ZnONPs and TiO_2_NPs adhering to the cell surface can generate extracellular ROS and induce intracellular generation of ROS, leading to oxidative stress on cells, distortion and damage of cell membranes, leakage of intracellular contents, and eventually cell death [21,50]. In this study, in vitro and in vivo actions of ZnO and TiO_2_ and their NPs on *D. dadantii* were processed in the dark; photo-oxidization and photocatalysis may not participate in the antibacterial mechanisms of ZnONPs and TiO_2_NPs against *D. dadantii*. ZnONPs and TiO_2_NPs may induce osmotic stress on bacterial cells in the dark and induce membrane depolarization and loss of membrane integrity, resulting in cellular leakage [51], as revealed by TEM. In addition, Zn^2+^ may be dissolved from ZnONPs and expose its heavy metal toxicity to cells.

The ZnONPs and TiO_2_NPs produced in this study show polymorphic structures and surface defects with numerous edges and pits. Such abrasive surface texture not only has more reactive surface sites but also tends to disrupt cell membranes and abrade biofilms. *D. dadantii* regulates plant surface colonization via regulation of flagella-mediated motility and biofilm formation. *D. dadantii* movement and attachment to plant surfaces and formation of biofilms on plant surfaces, in intercellular spaces, and in xylem vessels are essential for survival and completing disease cycles [52,53,54,55]. Perhaps, ZnONPs and TiO_2_NPs adhering to bacterial cell surfaces may inhibit bacterial movement and attachment to plant surfaces, inhibit bacterial growth, and damage bacterial cells and biofilms.

We previously produced AgNPs against *D. dadantii* by mixing AgNO_3_ solution with cell-free bacterial culture supernatants for 48 h [41]. That entire process required screening of bacterial culture supernatants and sterile operation to avoid contamination, which was time-consuming and not labor- and cost-effective as compared to this green-synthesis with the lemon fruit extract.

The reason we used lemon fruit extract to produce ZnONPs and TiO_2_NPs instead of AgNPs is to avoid using toxic Ag^+^. However, the green synthetic ZnONPs and TiO_2_NPs have properties distinct from ZnO and TiO_2_, such as the antibacterial activity against *D. dadantii*, and raise the concern about NP toxicity to plants and humans. NPs can be taken up and accumulated in plants and enter the food chains of animals and human, and thus pose a risk to human health [20,56,57].

ZnONPs and TiO_2_NPs may have positive and negative impacts on plants, depending on not only NP properties (size, shape, surface coating, and stability), concentration, and exposure time but also plant properties (susceptible or tolerant to NPs) and development stages. Generally, ZnONPs and TiO_2_NPs in excess are harmful to plants, while in traces can be beneficial for plants [56,57,58]. Bradfield et al. [59] grew sweet potato to maturity in field microcosms using substrate amended with either ZnONPs, CuONPs, or CeO_2_NPs or equivalent amounts of Zn^2+^, Cu^2+^, or Ce^4+^ at three concentrations (100, 500, or 1000 mg∙kg DW^−1^). Only the application with the highest concentration of Zn or Cu, which is unlikely to occur in the environment, caused adverse outcomes, that is, reduction in tuber biomass, metal accumulation, and dietary intake regardless of the chemical forms of the metals added to the substrate. The concentrations of metals were higher in the peels than in the inner tuber flesh of sweet potato. Under such conditions, metal oxide NPs pose no greater risk to sweet potato yield or food safety than do the ionic metals [59]. Bonilla-Bird et al. [60] immersed sweet potato tubers in suspensions or solutions of CuONPs, CuO, and CuCl_2_ at 25, 75, or 125 mg∙L^−1^ under continuous stirring for 30 min and found that the Cu concentration in internal tissues of tubers treated with CuONPs was similar to that in control tubers, suggesting no risk of Cu contamination in peeled tubers. The peel of sweet potato tubers restricts the inward radial transfer of metal NPs. Thorough cleaning of tuber surfaces and peeling of tubers can effectively reduce the consumer exposure to metal NPs or ionic metals [59].

## 5. Conclusions

We used ZnO and TiO_2_, which are generally recognized as safe substances, and extracts from edible lemon fruits, which are free of toxicants and rich in biomolecules, as safe raw materials to reduce health and environmental risks of the sources for the production of NPs. We developed a simple, rapid, cost-effective, and ecofriendly method to produce green ZnONPs and TiO_2_NPs simply by mixing ZnO or TiO_2_ solution with the lemon fruit extract. Actions of carboxylic acids, amino acids, and polyols in the lemon fruit extract may lead to the formation of the polymorphic ZnONPs and TiO_2_NPs. In contrast to ZnO and TiO_2_, the ZnONP and TiO_2_NP products effectively inhibited *D. dadantii* growth, swimming motility, biofilm formation, and maceration of sweet potato tubers and likely combat the bacterial pathogen via multiple mechanisms. The multiple bacteriocidal and bacteriostatic mechanisms would make it difficult for the pathogen to develop resistance to the NPs. The latently infected seed tuber is one of the major sources of the soft rot disease of sweet potato. The green ZnONPs and TiO_2_NPs appear to be promising materials to treat the seed tubers to avoid and reduce the pathogen contamination and to produce healthy crops. A study is needed to clarify if ZnONPs and TiO_2_NPs may contaminate the sweet potato tuber flesh and can be used to preserve and increase the shelf life of sweet potato tubers without exposing consumers to excess metals. More studies are needed before bringing the ZnONPs and TiO_2_NPs to the field to control the soft rot disease and promote sweet potato growth.
